# Human fasting modulates macrophage function and upregulates multiple bioactive metabolites that extend lifespan in *Caenorhabditis elegans*: a pilot clinical study

**DOI:** 10.1016/j.ajcnut.2022.10.015

**Published:** 2022-12-20

**Authors:** Christopher H. Rhodes, Chenghao Zhu, Joanne Agus, Xinyu Tang, Qianyan Li, JoAnne Engebrecht, Angela M. Zivkovic

**Affiliations:** 1 Department of Nutrition, University of California, Davis, Davis, CA; 2 Department of Molecular and Cellular Biology, University of California, Davis, Davis, CA

**Keywords:** prolonged fasting, fasting, intermittent fasting, innate immune system, anti-inflammatory, immunology, lifespan, longevity, fasting mimetic, macrophage

## Abstract

**Background:**

Periodic prolonged fasting (PF) extends lifespan in model organisms and ameliorates multiple disease states both clinically and experimentally owing, in part, to its ability to modulate the immune system. However, the relationship between metabolic factors, immunity, and longevity during PF remains poorly characterized especially in humans.

**Objective:**

This study aimed to observe the effects of PF in human subjects on the clinical and experimental markers of metabolic and immune health and uncover underlying plasma-borne factors that may be responsible for these effects.

**Methods:**

In this rigorously controlled pilot study (ClinicalTrial.gov identifier, NCT03487679), 20 young males and females participated in a 3-d study protocol including assessments of 4 distinct metabolic states: *1*) overnight fasted baseline state, *2*) 2-h postprandial fed state, *3*) 36-h fasted state, and *4*) final 2-h postprandial re-fed state 12 h after the 36-h fasting period. Clinical and experimental markers of immune and metabolic health were assessed for each state along with comprehensive metabolomic profiling of participant plasma. Bioactive metabolites identified to be upregulated in circulation after 36 h of fasting were then assessed for their ability to mimic the effects of fasting in isolated human macrophage as well as the ability to extend lifespan in *Caenorhabditis elegans.*

**Results:**

We showed that PF robustly altered the plasma metabolome and conferred beneficial immunomodulatory effects on human macrophages. We also identified 4 bioactive metabolites that were upregulated during PF (spermidine, 1-methylnicotinamide, palmitoylethanolamide, and oleoylethanolamide) that could replicate these immunomodulatory effects. Furthermore, we found that these metabolites and their combination significantly extended the median lifespan of *C. elegans* by as much as 96%.

**Conclusions:**

The results of this study reveal multiple functionalities and immunological pathways affected by PF in humans, identify candidates for the development of fasting mimetic compounds, and uncover targets for investigation in longevity research.

## Introduction

Periodic prolonged fasting (PF), defined here as ≥24 h of zero caloric intake, is one of the few known nutritional interventions capable of extending lifespan [[1], [2], [3], [4]]. These lifespan extending effects are linked to the reported efficacy of PF to modify the cellular processes of aging and help prevent, delay, or treat a range of diseases, particularly metabolic and chronic inflammatory diseases [1, 2, [5], [6], [7], [8], [9], [10]]. The mechanisms by which PF confers these beneficial health effects have been extensively reviewed [1, 2] and include the ability of PF to induce immunomodulatory effects on both the innate and adaptive immune systems [[11], [12], [13], [14], [15], [16]]. In innate immunity, PF has been shown to create effects on macrophage polarization [17, 18], inflammatory signaling pathways [14, 19], and monocyte-dependent autoimmune disease progression [16] without impairing appropriate responses to infection [16] or cancer [18]. This immunoregulation is of particular importance in mediating the role of PF in health and longevity as dysregulated innate immune function precedes and underlies the pathophysiology of a vast majority of chronic disease conditions including the damaging age-associated increase in chronic inflammation and neurodegenerative disease [11, 20, 21].

Despite the well-characterized link between PF and immunomodulation, there remains a paucity of research dedicated to investigating the underlying molecular mediators of these effects, especially in humans. One mechanism underlying these immunomodulatory effects may be the avoidance of the well-known proinflammatory effects of the postprandial state, which, although varied in magnitude between individuals, have been shown to be largely universal [22, 23]. Such evidence underscores that eating in and of itself disrupts immunological homeostasis and that overly frequent food consumption may induce damaging chronic inflammation. Although nutrition research has focused for years on the importance of food type and quantity in achieving health outcomes, the fundamental importance of when and how long to go without food has been neglected until recently. The biology of fasting is now starting to be uncovered and PF holds promise for the treatment and prevention of immune dysfunction [11]; however, there are multiple populations and individuals for whom prolonged periods between meals are contraindicated due to a variety of underlying conditions and who require alternative solutions to achieve the benefits of fasting. The identification of the molecular mediators of the beneficial immunoregulatory effects of PF is crucial for the discovery of compounds that can mimic the effects of fasting and enable populations that are precluded from fasting to achieve fasting-like benefits without the need to fast.

To address these knowledge gaps, we designed and performed a pilot clinical trial assessing 4 distinct nutritional states in each participant to determine the effects of 36 h of fasting on the plasma metabolome and macrophage functionalities of young healthy participants and discover potential molecular mediators of these effects.

## Methods

### Human clinical trial of PF

**Figure 1:**
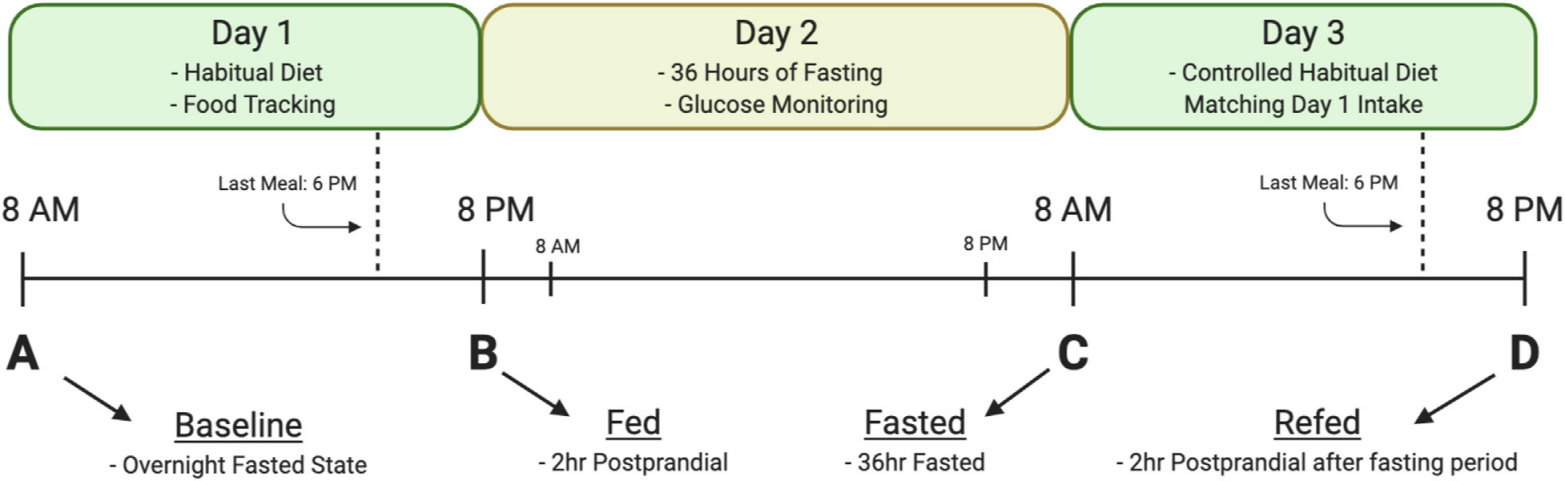
Timeline of 3-d human fasting trial: 20 participants underwent a 3-d clinical trial consisting of 4 study visits in 4 distinct nutritional states to allow for the sensitive assessment of the effects of 36 h of fasting versus an overnight fasted state and the carryover effects of fasting onto the next eating day. On day 1, participants provided an overnight fasting baseline blood sample (A) and then went about their normal routine and habitual diet while tracking their food intake until 18:00 where participants ate their last meal. A 2-h postprandial fed blood sample was taken at 20:00 on day 1 (B), after which participants began their 36-h fast while being monitored for compliance via glucose monitors. At 08:00 on day 3, participants provided a 36-h fasted blood draw (C) and then were given a copy of their diet record from day 1 and instructed to eat the identical diet as they recorded on day 1. At 18:00 on day 3, participants ate their last meal and received a final 2-h postprandial re-fed blood draw (D) concluding the study.

To assess the effects of PF on the plasma metabolome and macrophage functionalities of human participants, we performed a 3-d human study of a single bout of 36 h of fasting in 20 young healthy participants (see Figure 1). Full clinical study protocols can be found at clinicaltrials.gov (NCT03487679) and were approved by the UC Davis Institutional Review Board (IRB) (IRB identification [ID], 918915-4). All participants provided informed consent before enrolling in study protocols, and all participants were recruited and completed study protocols between April and May of 2018. The full inclusion and exclusion criteria can be found at clinicaltrials.gov (NCT03487679). Briefly, participants were included if they were aged 20–40 y, had a body mass index (BMI) within the range of 19–27 kg/m^2^, had fasting glucose levels in the clinically normal range of 70–100 mg/dL, had no documented health conditions, and had no extreme dietary or exercise patterns. Seventy-two interested individuals were screened for eligibility, and 20 met the inclusion and exclusion criteria and were enrolled in the study (see Supplemental Figure 1). There were no dropouts, adverse events, or protocol violations, and all 20 participants successfully completed the 3-d trial and were included in experimental analysis (see Supplemental Figure 1). All clinical study activities were performed at the UC Davis Ragle Human Nutrition Research Center. On day 1, participants in an overnight (12 h) fasted state provided a baseline blood draw at approximately 08:00 representing the baseline state and were then instructed to go about their normal routine and, importantly, consume their habitual diet while keeping track of their dietary intake using a detailed food record, which was confirmed through a 24-h dietary recall with trained study personnel. Participants were instructed to eat their last meal at 18:00 on day 1 and then returned to the Ragle Center for a 2-h postprandial blood draw at 20:00 representing the fed state. Participants then underwent a period of 36 h of fasting representing the rest of day 1 and all of day 2 during which they were monitored for compliance utilizing glucose meters. On day 3 at approximately 08:00, participants provided a 36-h fasted blood draw representing the fasted state and were then given a copy of their recorded dietary intake from day 1. Participants were then instructed to eat exactly the same diet as they recorded on day 1 throughout all of day 3 and, as before, ate their last meal at 18:00 and received a final 2-h postprandial blood draw representing the re-fed state. Throughout the study protocols, participants were advised to maintain their habitual water consumption and daily routines with the exception of strenuous exercise. To our knowledge, this is the first study to utilize this methodology of what we term a “controlled habitual diet” in monitoring and controlling food intake during a clinical study. A controlled habitual diet as we implemented here offers several advantages to nutritional studies, particularly crossover studies where each participant acts as their own control. Importantly, it allows researchers to control for nutritional intake between states while avoiding the well-known disruptive metabolic effects of introducing a novel standardized diet into disparate human populations [24]. Unlike other fasting trials, utilizing a controlled habitual diet as well as assessing 4 distinct nutritional states from each individual throughout the course of the study allowed for a clear comparison of not only the postprandial state to the 36-h fasted state but also the assessment of the distinct effects of PF beyond a typical overnight fast and the potential carryover effects of PF to a re-fed postprandial state after fasting.

### Blood processing

All participant blood samples were collected in ethylenediaminetetraacetic acid plasma tubes and immediately centrifuged (1500 × *g*, 10 min, 4°C), portioned into aliquots, and stored at −80°C until sample analysis.

### NMR LipoProfile

Plasma lipoprotein particle sizes and concentrations were analyzed by proton nuclear magnetic resonance (NMR) spectroscopy at LabCorp (LipoScience, Inc.). This analysis, certified according to the Clinical and Laboratory Standards Institute EP5-A2 guidelines, uses NMR to estimate the number and size of lipoprotein particles within the lipoprotein subclasses, including very-low-density lipoprotein, low-density lipoprotein (LDL), and high-density lipoprotein (HDL) particles from small to large, reporting a calculated lipoprotein insulin resistance index [25], which has been shown to be highly correlated with multiple indices of insulin resistance and predictive of incident type 2 diabetes across large multiethnic cohort studies [[25], [26], [27]]. Included in the report are total cholesterol, LDL cholesterol, HDL cholesterol, and triglycerides calculated from the NMR spectral data. An additional result from this test panel includes GlycA, a measurement of NMR signal from acute-phase proteins, which measures the overall degree of inflammation in plasma and has been shown to be associated with incident cardiovascular disease, cardiometabolic risk, and a number of inflammatory conditions, including rheumatoid arthritis [28, 29]. Finally, the test panel also provides concentrations of ketone bodies, glucose, and total protein.

### Metabolomic analyses

Metabolomic analysis was conducted at Metabolon, Inc. as previously described [30]. Briefly, samples were homogenized and subjected to methanol extraction and then split into aliquots for analysis by ultrahigh performance liquid chromatography/mass spectrometry (MS) in the positive (2 methods) and negative (2 methods) modes. The positive mode included positive ionization chromatographically optimized for hydrophilic compounds (liquid chromatography-tandem mass spectrometry [LC–MS/MS] Pos Polar) and positive ionization chromatographically optimized for hydrophobic compounds (LC–MS/MS Pos Lipid). The negative mode included negative ionization optimized conditions (LC–MS/MS Neg) and negative ionization with hydrophilic interaction liquid chromatography (LC–MS/MS Polar). All chromatography was performed using a Waters Acquity UPLC (Waters) held at 40°C–50°C. The MS methods utilized Thermo Scientific Q Exactive high-resolution/accurate mass spectrometers with heated electrospray ionization (HESI-II) sources and Orbitrap mass analyzers (Thermo Fisher Scientific) operated at 35,000 mass resolution. Metabolites were then identified by automated comparison of ion features to a reference library of chemical standards followed by visual inspection for quality control as previously described [31]. For statistical analyses and data display, any missing values were assumed to be below the limits of detection; these values were imputed with the compound minimum (minimum value imputation).

### Primary macrophage isolation

Primary macrophages for use in experimental in vitro assays of participant plasma functionalities were isolated from healthy male volunteers in an overnight fasted state. Peripheral blood mononuclear cells were isolated using Ficoll gradient extraction (Sigma, GE17-1440-03) and then cultured in flasks with Roswell Park Memorial Institute Medium 1640 (RPMI) (Thermo Fisher Scientific, 11875119) and 1x penicillin–streptomycin–glutamine (1xPSG) (Thermo Fisher Scientific, 11875119) for 3 h to induce adhesion. Nonadherent cells were discarded, and adherent cells were placed into RPMI, 10% fetal bovine serum (FBS) (Thermo Fisher Scientific, A3160402), and 1xPSG containing 20 ng/mL of human macrophage colony-stimulating factor (Peprotech, 300-25) for 7 d to induce macrophage differentiation.

### THP-1 macrophage differentiation

For certain assays requiring high cellular concentrations beyond what was feasible to isolate from human volunteers, THP-1 monocytes (ATCC, TIB-202) were cultured in RPMI 1640, 10% FBS, and 1xPSG and differentiated into M0 macrophage using 100-nM phorbol 12-myristate 13-acetate (Sigma, P8139) for 2 d followed by incubation with clean RPMI, 10% FBS, and 1xPSG for 1 d.

### Functional analyses of participant plasma and isolated metabolites

Analyses of molecular functionalities of participant plasma from each timepoint and in vitro effects of participant plasma and isolated metabolites on human macrophage functionalities and activities were performed as described in the following. All functional analyses were performed in duplicate for each experimental sample assessed.

#### Citrullinated fibrinogen immune complex assay

The responsiveness of treated primary macrophage to citrullinated fibrinogen immune complex (cFb IC), an Fc-γ receptor stimulant and in vitro model of autoimmune disease [32], was performed as previously described [32]. Briefly, 20 μg/mL of citrullinated fibrinogen was plated onto 96-well plates, blocked, and treated with 20 μg/mL of antifibrinogen antibody (Agilent, A008002-2) to form cFb ICs. Primary human macrophage in RPMI 1640 and 1xPSG were treated with participant plasma to a final concentration of 20%, isolated metabolites, or FBS to a final concentration of 20% as a positive control for 1 h and then plated at 5.0 × 10^5^ cells/mL and allowed to incubate at 37°C overnight (∼18 h). A negative control well containing macrophage in RPMI 1640, 20% FBS, and 1xPSG without immune complexes was also included. Tumor necrosis factor alpha (TNF-α) secretion from treated macrophage was then measured in the cell supernatant via enzyme-linked immunosorbent assay (PeproTech, 900-K25). After obtaining the results from our initial experiment of participant plasma on macrophage reactivity in this model using a single primary cell donor, we performed a follow-up experiment to confirm our observed results in a second primary cell donor. From those results, we found the same significant effects of participant plasma on macrophage reactivity from both primary cell donors (see Supplemental Figure 2) and, thus, proceeded to use macrophage from a single consistent cell donor for the remainder of the analyses utilizing primary macrophage.

#### Antioxidant capacity

Plasma antioxidant capacity was assessed using a commercially available kit (Abcam, ab65329).

#### Cholesterol efflux ability

Cholesterol efflux ability of participant plasma from primary macrophage was measured using a commercially available kit (Abcam, ab196985) following the manufacturer’s instructions with the following modifications: primary macrophages were lipid loaded with one-half of the recommended labeled cholesterol for 4 h prior to incubation with either participant plasma in equilibration buffer to a final concentration of 2% or FBS in equilibration buffer to a final concentration of 2%. Cholesterol efflux from macrophage was measured after 2 h of incubation with participant plasma.

#### Intracellular reactive oxygen species assay

The effect of treatment with participant plasma at a final concentration of 20% and individual metabolites on cellular reactive oxygen species (ROS) accumulation in primary macrophage was assessed using a commercially available kit according to the manufacturer’s instructions (Abcam, ab113851).

#### Cyclooxygenase activity assay

The effect of treatment with participant plasma and individual metabolites on the total cellular cyclooxygenase (COX) activity of primary macrophage was measured using a commercially available kit (Cayman Chemical, 760151). To induce COX-2 expression, primary macrophages were incubated with 10 ng/mL of lipopolysaccharide (LPS) (Sigma, LPS25) in RPMI and 1xPSG along with 20% participant plasma or in RPMI, 20% FBS, and 1xPSG either alone as a positive control or with isolated metabolites overnight (∼18 h) before measuring total COX activity from cell lysates. A negative control of macrophage in RPMI, 20% FBS, and 1xPSG without LPS was also performed.

#### M1 polarization assay

The effect of treatment with participant plasma and individual metabolites on the induction of M1 polarization was assessed via enzymatic analysis of nitric oxide synthase (NOS) and arginase activity in macrophage cell lysates. M0 THP-1 macrophages were incubated for 2 d with 100 ng/mL of LPS and 20 ng/mL of interferon gamma (INF-γ), a known M1 polarization inducer [33], in either RPMI 1640 and 1xPSG with 20% participant plasma or RPMI, 20% FBS, and 1xPSG alone as a positive control or with isolated metabolites. A negative control of macrophage in RPMI, 20%, and 1xPSG without LPS and INF-γ was also performed. NOS and arginase activities of these cells were assessed in cell lysates standardized to total protein content via commercially available kits according to the manufacturer’s instructions (Abcam, ab211083; ab180877).

### *Caenorhabditis elegans* lifespan studies

*Caenorhabditis elegans* var. Bristol (N2) was used as the wild-type strain. Strains were maintained, and lifespan assays were performed at 20°C. Twenty-four hours after seeding *Escherichia coli* (OP50) on standard Nematode Growth Medium (NGM) plates, the bacteria were killed by 4-min exposure to ultraviolet (UV) irradiation using a Stratalinker UV crosslinker (Stratagene, Model 2400). The following concentrations of compounds were used: *1*) 0.2 mM of spermidine, *2*) 0.5 mM of 1-methylnicotinamide (1-MNA), *3*) 0.01 mM of palmitoylethanolamide (PEA), and *5*) 0.01 mM of oleoylethanolamide (OEA). The spermidine and 1-MNA concentrations were selected based on previous research showing lifespan extension effects at 0.2 and 0.5 mM, respectively [34, 35], whereas the PEA and OEA concentrations were chosen based on their efficacy during our in vitro analyses. Spermidine and 1-MNA were diluted into 100 μL of sterilized water and applied to the top of the agar medium (3-mL NGM plates). Plates were then gently swirled to allow compounds to spread to the entire NGM surface. Identical solutions of compound-free water were used for the control plates. Plates were then allowed to dry overnight. The procedure was repeated each time worms were transferred to fresh plates (every 2–4 d). PEA and OEA were added to cooled agar, prior to solidification; for the combination, all compounds were added to cooled agar and plates stored at 4°C. Synchronous worm populations were generated by hypochlorite treatment of gravid adults, and lifespan assays were performed beginning at the L4 stage (day 0). Eight plates of 15 worms each were exposed to the indicated compounds. Animals were transferred to fresh plates every 2 d for the first week and then 3–4 d thereafter. Worms were examined for touch-provoked movement and pharyngeal pumping until death. Worms that died due to desiccation from crawling on the edge of the plates were censored.

## Statistical analysis

### Metabolomic analyses

All statistical analyses were performed in the statistical language R (3.6.1). A linear model was used to test the differences between experimental groups by giving each subject an individual intercept using the R package limma [36]; *P* < 0.05 was considered significant. Multiple comparisons were corrected using the Benjamini–Hochberg method. The MS intensity of each metabolite was log transformed prior to group comparisons. Fisher’s exact test was performed to test whether metabolites that were significantly increased or decreased (*P* < 0.05) were enriched in particular pathways using Metabolon’s Portal Database.

### In vitro functional assessments

An analysis of variance (ANOVA) mixed model was fit for each in vitro functional assay using the treatment group as a fixed variable and subject ID as a random variable. The ANOVA model was fit using the R package lme4 (1.1.21) [37]. Post hoc comparison was performed on the fitted ANOVA model using Tukey’s all-pair comparisons with the R package multcomp (1.4.13) [38].

### C. elegans lifespan analysis

The Kaplan–Meier survival curves were created to evaluate *C. elegans* lifespan. The R package survival (3.1.8) was used to fit the Cox proportional hazards regression model to evaluate differences in survival between each treatment group and control. The median lifespan is given as the length in days at which each group reached 50% survival, and the maximal lifespan was calculated as the mean lifespan of the longest lived 10% of organisms for each group. The n of each experimental group was 120.

## Results

### PF significantly alters clinical markers of metabolic health

To assess the effects of PF on the metabolome and macrophage functionalities of human participants, we performed a 3-d human study of 36 h of fasting in 20 young healthy participants (age, 27.5 ± 4.35 y; BMI, 24.1 ± 2.66 kg/m^2^; male, *n* = 10; female, *n* = 10). Across the trial, plasma was collected during 4 distinct nutritional states, including an overnight fasted baseline state (baseline), a 2-h postprandial state (fed), a 36-h fasted state (fasted), and a second 2-h postprandial state collected 12 h after the 36-h fasting period (re-fed). A timeline of the 3-d study protocol is shown in Figure 1, and the baseline characteristics of participants are shown in Table 1. Of 20 participants enrolled, all successfully completed the study protocol without any protocol violations and were included in experimental analyses (see Supplemental Figure 1). No significant differences were observed between the mean dietary intakes of participants during day 1 or 3 of the study indicating compliance to the controlled habitual diet protocol (see Supplemental Table 1). Compliance to fasting was assessed through the use of personal glucose monitoring throughout the waking hours of the fasting period as well as assessment of elevated ketone body levels in the fasted state (see Table 2, Supplemental Figure 3). All participants were found to be compliant to the fasting period as indicated by elevated ketone body levels in the fasted state above the values of the baseline state (see Table 2) and consistent glucose readings below 100 mg/dL throughout the fasting period (see Supplemental Figure 3). Typical of fasting, NMR LipoProfile data from each timepoint showed significantly increased levels of circulating ketone bodies (2284 ± 1261 vs. 191.6 ± 129.9 μM, *P* < 0.001) and amino acids (561.1 ± 33.2 vs. 530.2 ± 52.8 a.u., *P* < 0.001) along with significantly decreased glucose values (74.5 ± 9.1 vs. 86.4 ± 11.4 mg/dL, *P* < 0.001) in the fasted versus the baseline state, respectively (see Table 2). Similarly, the circulating triglyceride levels (84 ± 28 vs. 118.6 ± 49.3 mg/dL, *P* < 0.001) significantly decreased, and the ketone body levels (353.1 ± 334.4 vs. 187.1 ± 160.6 μM, *P* < 0.001) and glucose levels (97.1 ± 10.3 vs. 88.4 ± 14.1 mg/dL, *P* < 0.014) significantly increased in the re-fed state versus the fed state, respectively, indicating that even after a full day of eating, there are still metabolic carryover effects of PF (see Table 2). Interestingly, the LDL cholesterol levels also significantly increased in the fasted state versus the baseline state (85.8 ± 25.1 vs. 77.5 ± 27 mg/dL, *P* < 0.001), whereas the HDL cholesterol levels were unchanged (67.7 ± 15.1 vs. 66.1 ± 16.8 mg/dL, *P* < 0.622) indicating potentially altered lipoprotein and cholesterol metabolism in response to PF (see Table 2).

**Table 1:** Baseline characteristics of the study participants

Participant characteritic	Value at baseline
Age (y)	27.5 ± 4.35
Height (cm)	170.7 ± 9.8
Weight (kg)	71.3 ± 13.4
BMI	24.3 ± 3.1
Waist circumference (cm)	78.8 ± 8.9
Systolic blood pressure (mmHg)	113.7 ± 9
Diastolic blood pressure (mmHg)	71.6 ± 5.5

Average characteristics of all 20 study participants at baseline.

**Table 2:** Nuclear magnetic resonance LipoProfile data of the study participants

Participant characteristic	Baseline (A)	Fed (B)	Fasted (C)	Re-fed (D)	pval_AC	pval_BD
HDL cholesterol (mg/dL)	66.1 ± 16.8	68.2 ± 17.5	67.7 ± 15.1	67.5 ± 15.9	0.622	0.948
LDL cholesterol (mg/dL)	77.5 ± 27	76.8 ± 29.4	85.8 ± 25.1	82.2 ± 30.1	< 0.001	0.058
Triglyceride (mg/dL)	94.4 ± 31.6	118.6 ± 49.3	81.8 ± 17.8	84 ± 28	0.091	< 0.001
Total cholesterol (mg/dL)	163.1 ± 36.3	168 ± 39.2	166.8 ± 29.2	165.2 ± 37	0.417	0.594
Glucose (mg/dL)	86.4 ± 11.4	88.4 ± 14.1	74.5 ± 9.1	97.1 ± 10.3	< 0.001	0.014
Ketone bodies (μmol/L)	191.6 ± 129.9	187.1 ± 160.6	2284 ± 1261	353.1 ± 334.4	< 0.001	< 0.001
Amino acids (a.u.)	530.2 ± 52.8	537.5 ± 47.7	561.1 ± 33.2	550.9 ± 43	< 0.001	0.129
LPIR^1^	19.8 ± 13.5	22.9 ± 13.1	12.7 ± 5.6	16.4 ± 8.7	0.166	0.161
GlycA (μmol/L)	329.9 ± 54.9	325 ± 48.6	316.4 ± 43.9	317.6 ± 50	0.154	0.318

Values are presented as mean ± standard deviation. *n* = 20. Average nuclear magnetic resonance LipoProfile data from 20 study participants across timepoints. Significance was determined using Tukey’s test, and significance values are given comparing the baseline state (A) with the fasted state (C) and fed state (B) with the re-fed state (D). Elevated ketone body levels in the fasted versus the baseline indicate compliance to fasting. LPIR, Lipoprotein insulin resistance index.

1 LPIR index score from 0 to 100.

### PF enhances plasma functionalities and induces anti-inflammatory effects in macrophage

**Figure 2:**
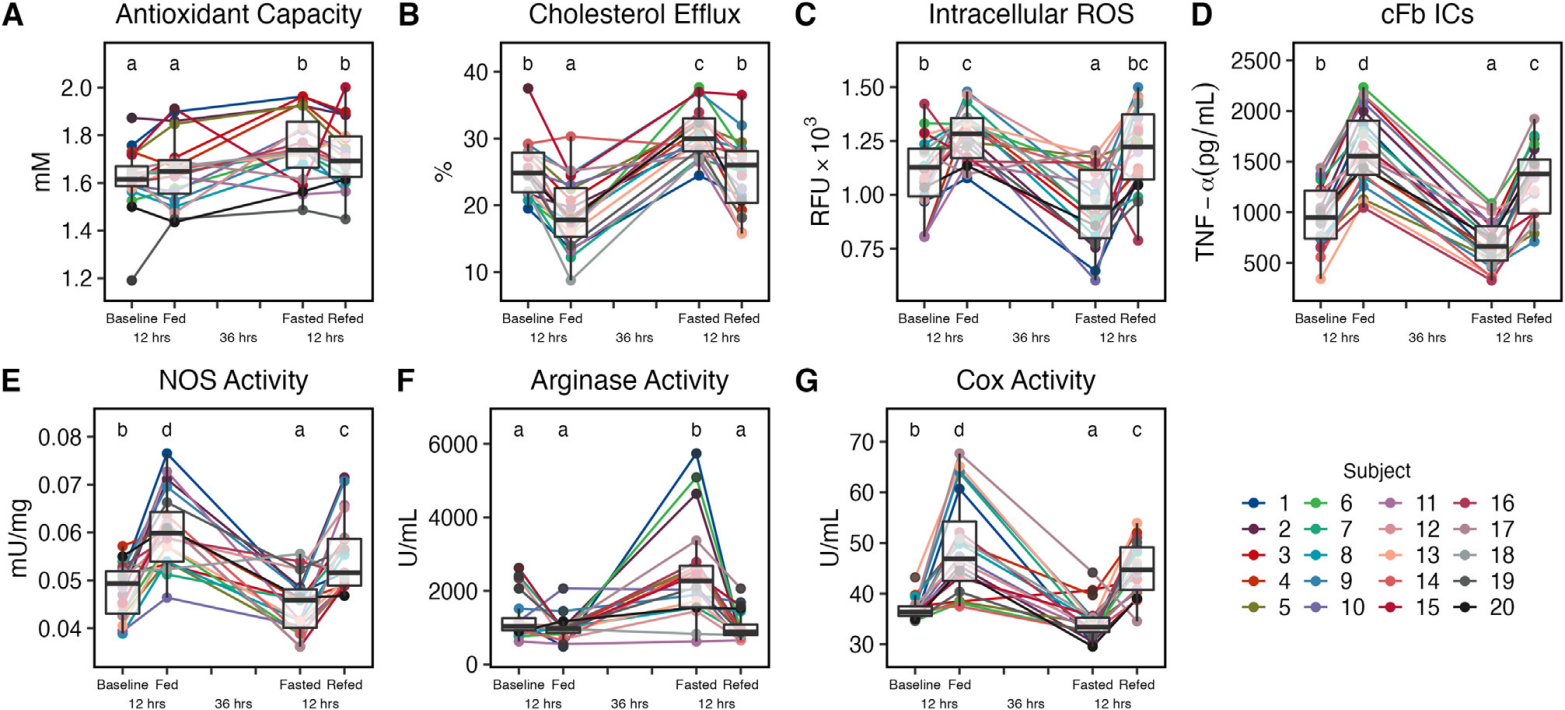
Prolonged fasting significantly alters the effects of participant plasma on macrophage. In vitro analysis of the total antioxidant capacity of participant plasma (A). Significance between timepoints for all analyses (A–G) is denoted by their assigned letter (a–d) where groups that share an assigned letter in common were found to have no significant differences (*P* > 0.05) and groups that do not share an assigned letter in common were found to be significantly different (*P* < 0.05) from each other. The significance levels are provided for the baseline versus fasted states and fed versus re-fed states where significance (*P* < 0.05) is achieved. Significance between states was determined using Tukey’s test. Baseline versus fasted states (1.61± 0.13 vs. 1.75 ± 0.14 mM, *P* < 0.0001) and fed versus re-fed states (1.66 ± 0.14 vs. 1.71 ± 0.13 mM, *P* = 0.034). In vitro analysis of the cholesterol efflux capacity of participant plasma from lipid-loaded primary human macrophage (B). Baseline versus fasted states (25.3% ± 4.1% vs. 30.7% ± 3.7%, *P* < 0.0001) and fed versus re-fed states (18.6% ± 5.2 vs. 24.7% ± 5.5%, *P* < 0.0001). In vitro analysis of intracellular ROS production in hydrogen peroxide-stimulated primary human macrophage treated with participant plasma (C). Baseline versus fasted states (1107 ± 176 vs. 948 ± 186 RFU, *P* = 0.0018). In vitro analysis of tumor necrosis factor alpha (TNF-α) secretion from citrullinated fibrinogen immune complex (cFb IC)-stimulated primary human macrophage treated with participant plasma (D). Baseline versus fasted states (964 ± 304 vs. 677 pg/mL ± 232, *P* = 0.0003) and fed versus re-fed states (1626 ± 371 vs. 1313 ± 352 pg/mL, *P* = 0.0004). In vitro analysis of NOS activity in lipopolysaccharide (LPS)- and interferon gamma (INF-γ)-stimulated THP-1 macrophage treated with participant plasma (E). Baseline versus fasted states (0.048 ± 0.005 vs. 0.045 ± 0.006 mU/mg, *P* = 0.035) and fed versus re-fed states (0.06 ± 0.008 vs. 0.055 ± 0.008 mU/mg, *P* = 0.017). In vitro analysis of arginase activity in LPS- and INF-γ- stimulated THP-1 macrophage treated with participant plasma (F). Baseline versus fasted states (1275 ± 594 vs. 2494 ± 1340 U/mL, *P* < 0.0001). In vitro analysis of total cyclooxygenase (COX) activity in LPS-stimulated primary macrophage treated with participant plasma (G). Baseline versus fasted states (36.8 ± 2.1 vs. 34.3 ± 3.6 U/mL , *P* = 0.013) and fed versus re-fed states (49.4 ± 9.9 vs. 44.9 ± 5.2 U/mL, *P* = 0.034). Each analysis described earlier was performed using plasma samples from all 20 study participants. Assays were performed in duplicate using primary human macrophage from a single healthy donor (A–D) or differentiated THP-1 macrophage (E–G). Boxplots displayed on graphs show the median, first quartile, third quartile, minimum, and maximal values of each timepoint.

To assess the molecular effects of PF on participant plasma, we performed multiple biochemical assessments of the functionality of participant plasma across states as well as the effect of participant plasma on the functionalities of primary human macrophage using a novel ex vivo modeling technique. For all assessments reported, we found significant differences between not only the fed and fasted states but also the baseline and fasted states, indicating the unique effects of PF that are not achieved through an ordinary overnight fast. First, we found that PF was capable of significantly increasing the total antioxidant capacity of human plasma compared with the baseline state (1.75 ± 0.14 vs. 1.61 ± 0.13 mM, *P* < 0.0001) and strikingly that that this effect was also achieved in the re-fed state compared with that in the fed state (1.71 ± 0.13 vs. 1.66 ± 0.13 mM, *P* = 0.034), indicating a powerful carryover effect of PF to the re-fed postprandial state even after a full day of habitual eating (see Figure 2A). Similarly, we found that the ability of participant plasma to efflux cholesterol from lipid-loaded macrophage in the fasted state also significantly increased from the baseline and fed states (30.7% ± 3.7% vs. 25.3% ± 4.1%, *P* < 0.0001, vs. 18.6% ± 5.2%, *P* < 0.0001) and that the re-fed state had significantly higher efflux ability than the fed state (24.7% ± 5.5% vs. 18.6% ± 5.2%, *P* < 0.0413) (see Figure 2B). We also found that treatment of primary human macrophages with participant plasma was capable of significantly decreasing cellular ROS accumulation in the fasted state compared with that in the baseline and fed states (948 ± 186 vs. 1107 ± 176 RFU, *P* = 0.0018, vs. 1275.8 ± 118.8 RFU, *P* < 0.0001, respectively) (see Figure 2C).

As previous studies have shown immunomodulatory effects of PF on macrophage [14, 18, 39] and monocyte-driven autoimmune disease [16], we assessed the effects of participant plasma on primary human macrophages in a previously described in vitro model of autoimmune disease [32]. Remarkably, we found a highly significant decrease in proinflammatory TNF-α secretion from primary human macrophage treated with fasted plasma compared with that from primary human macrophage treated with baseline and fed plasma during stimulation with cFb ICs (677 ± 232 vs. 964 ± 304 pg/mL, *P* = 0.0003, vs. 1626 ± 371 pg/mL, *P* < 0.0001), and this effect carried over to the re-fed state compared with that in the fed state (1313 ± 352 vs. 1626 ± 371 pg/mL, *P* = 0.0004) (see Figure 2D). To further investigate the scope of the observed immunomodulation, we assessed the effects of treatment of THP-1 macrophages with participant plasma during in vitro induction of M1 polarization [33]. We found that treatment with participant plasma from the fasted state versus the fed and baseline states significantly decreased cellular NOS activity (0.045 ± 0.006 vs. 0.06 ± 0.008 mU/mg, *P* < 0.0001, vs. 0.048 ± 0.005 mU/mg, *P* = 0.035) whose activity is highly associated with classical activation and M1 polarization [33] along with concomitant increases in arginase activity, whose activity is highly associated with alternative activation and M2 polarization [33], in fasted versus baseline and fed plasma-treated cells (2494 ± 1340 vs. 1275 ± 594 U/mL, *P* < 0.0001, vs. 980 ± 332 U/mL, *P* < 0.001, respectively) (see Figure 2E–F). In agreement with previous mouse studies of PF and macrophage polarization [18, 39], these results indicate that treatment with fasted plasma is also capable of affecting the polarization state of macrophage in humans. Furthermore, that fasted plasma is capable of modulating cells away from classical activation responses and toward alternative activation responses.

To further elucidate the cellular pathways that may be involved in these immunomodulatory effects, we investigated the effects of treatment with participant plasma on the total COX activity of primary macrophages during LPS stimulation. We found that treatment with fasted plasma was able to significantly decrease total COX activity versus the baseline and fed plasma-treated cells (34.3 ± 3.6 vs. 36.8 ± 2.1 U/mL, *P* = 0.013, vs. 49.4 ± 9.8 U/mL, *P* < 0.0001, respectively), indicating a previously unknown role of COX signaling in PF-induced immunomodulation (see Figure 2G). These results show that PF significantly alters the functionality of human plasma, the anti-inflammatory effects of PF are inducible even in nonfasted macrophage through treatment with participant plasma in a 36-h fasted state, and these effects are quantitatively larger than a typical overnight fast. These results suggested that there are numerous plasma-borne factors that are differentially regulated between the baseline and fasted states that mediate the enhanced functional effects observed in the fasted plasma. To identify these factors, we performed comprehensive metabolomics on each participant plasma sample across all 4 nutritional states.

### PF robustly and uniquely alters the human plasma metabolome

**Figure 3:**
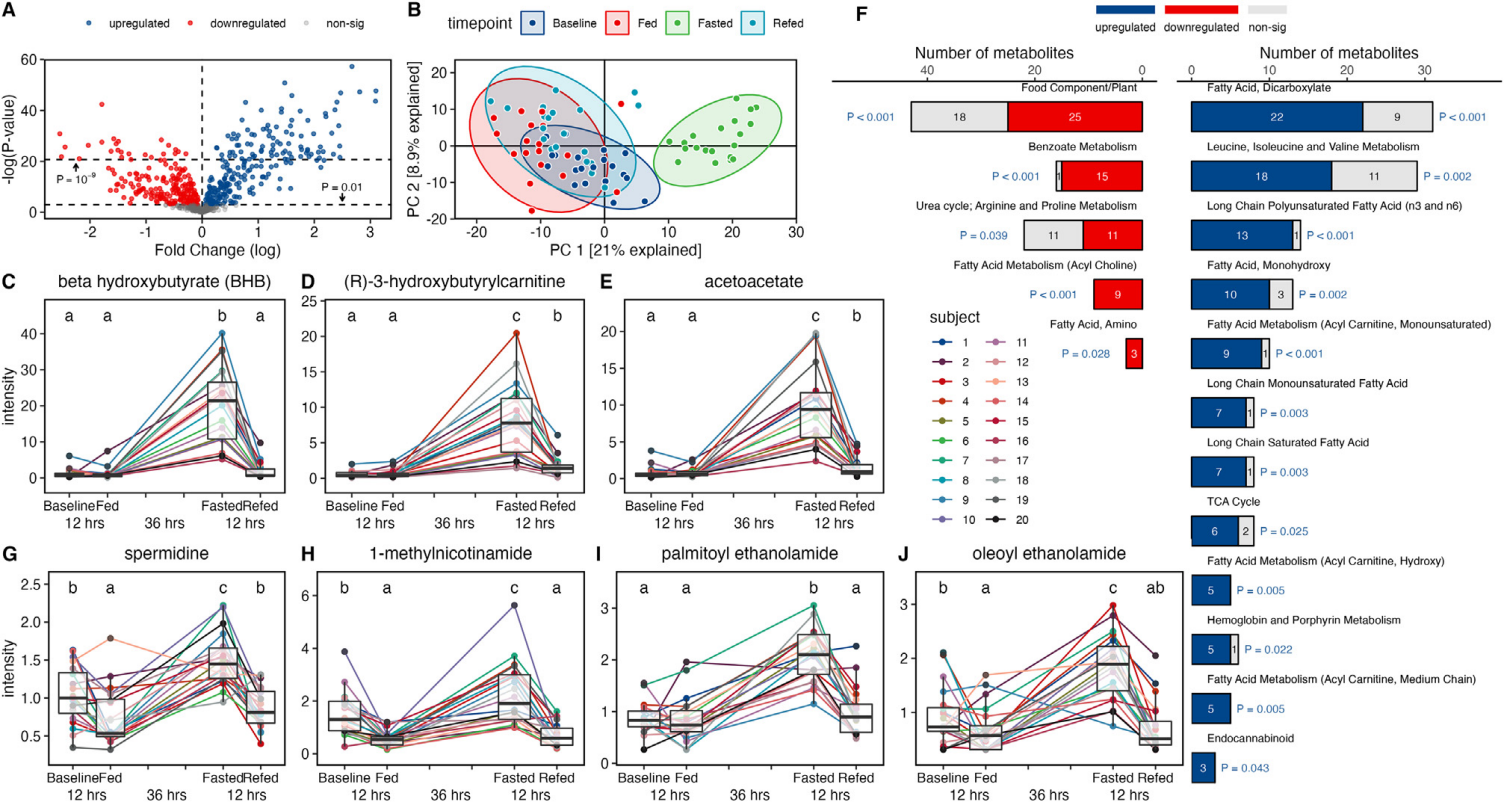
Prolonged fasting dramatically alters human metabolome and upregulates bioactive metabolites. Volcano plot analysis of the fasted metabolite levels compared with the baseline metabolite levels showing over 375 significantly different metabolites between the 2 states (A). Principal component analysis of complete metabolite datasets between the baseline, fed, fasted, and re-fed states (B). Circulating levels of the ketone body beta-hydroxybutyrate (BHB) in participant plasma (C). Significance between timepoints for all metabolite levels (C–E, G–J) is denoted by their assigned letter (a–c) where groups that share an assigned letter in common were found to have no significant differences (*P* > 0.05) and groups that do not share an assigned letter in common were found to be significantly different (*P* < 0.05) from each other. The significance levels are provided for the baseline versus fasted states and fed versus re-fed states where significance (*P* < 0.05) is achieved. Baseline versus fasted (1.16 ± 1.36 vs. 19.80 ± 10.80 RA, *P* < 0.0001). Circulating levels of (R)-3-hydroxybutyrylcarnitine in participant plasma (D). Baseline versus fasted states (0.57 ± 0.45 vs. 7.98 ± 5.10 RA, *P* < 0.0001) and fed versus re-fed states (0.60 ± 0.60 vs. 1.59 ± 1.34, *P* < 0.0001). Circulating levels of the ketone body acetoacetate in participant plasma (E). Baseline versus fasted states (0.75 ± 0.86 vs. 9.89 ± 5.37, *P* < 0.0001) and fed versus re-fed states (0.78 ± 0.63 vs. 1.52 ± 1.32, *P* = 0.025). Pathway analysis of metabolic datasets showing significantly differentially regulated pathways between the baseline and fasted states determined using Fisher’s exact test (F). Circulating levels of spermidine in participant plasma (G). Baseline versus fasted states (1.03 ± 0.37 vs. 1.51 ± 0.35, *P* = 0.0001) and fed versus re-fed states (0.74 ± 0.37 vs. 0.87 ± 0.27, *P* = 0.025). Circulating levels of 1-methylnicotinamide (1-MNA) in participant plasma (H). Baseline versus fasted states (1.52 ± 0.84 vs. 2.28 ± 1.19, *P* = 0.0038). Circulating levels of palmitoylethanolamide (PEA) in participant plasma (I). Baseline versus fasted states (0.87 ± 0.30 vs. 2.06 ± 0.51, *P* < 0.0001). Circulating levels of oleoylethanolamide (OEA) in participant plasma (J). Baseline versus fasted states (0.93 ± 0.46 vs. 1.83 ± 0.61, *P* < 0.0001). The n of each analysis described earlier was 20. Significance for Figure A–E and G–J was determined using a linear fit model. Moderated t-statistics were computed by empirical Bayes moderation, and *P* values were adjusted using the Benjamini–Hochberg method. Significance was determined using Fisher’s exact test for Figure F. Boxplots displayed on graphs show the median, first quartile, third quartile, minimum, and maximal values of each timepoint.

Comprehensive untargeted metabolomic analysis of participant plasma revealed remarkable alterations to the human metabolome in response to feeding, fasting, and re-feeding (see Figure 3). However, to delineate the distinct effects of PF rather than those achieved by the resolution of the postprandial response, we focused our analysis here on differences observed between the overnight fasted baseline state and the 36-h fasted state. Even between the baseline and fasted states, we found >375 significantly differentially regulated metabolites even after adjustments for multiple comparisons (see Figure 3A). Principal component analysis revealed a distinct metabolome of the fasted state that was different from and completely nonoverlapping with all other states (see Figure 3B). Moreover, we found significant and universal responses in multiple metabolites during PF as highlighted by our data of the 3 metabolites with the highest fold change in relative abundance in response to fasting including the known anti-inflammatory ketone body beta-hydroxybutyrate (BHB) [40] (1.16 ± 1.36 vs. 19.8 ± 10.8, *P* < 0.0001), 3-hydroxybutyrylcarnitine (0.57 ± 0.45 vs. 7.98 ± 5.10, *P* < 0.0001), and acetoacetate (0.75 ± 0.86 vs. 9.89 ± 5.37, *P* < 0.0001), indicating multiple obligate responses to acute fasting in humans (see Figure 3C–E). However, even within these obligate responses, there was also considerable interindividual variability in the magnitude of response between participants by as much as 14 times between the highest and lowest levels of an individual metabolite between participants in the fasted state (e.g., 3-hydroxybutyrylcarnitine) (see Figure 3D). Pathway analysis of the most differentially affected pathways between the baseline and fasted states revealed alterations to multiple fatty acid and amino acid biosynthesis and degradation pathways, ketone body metabolism, tricarboxylic acid cycle, and nicotinamide metabolism (see Figure 3F). In agreement with a recent trial of long-term human alternate day fasting [13], this study also found highly increased levels of immunomodulatory polyunsaturated fatty acids in the fasted state, indicating the potential involvement of altered fatty acid and eicosanoid signaling in the mechanisms underlying the effects of fasted plasma on macrophage functionality (see Figure 3F). Ultimately, PF induced widespread, highly significant, and, in several cases, universal alterations to the human metabolome beyond what is achieved during a typical overnight fast. Given these stark modulations, we sought to assess how differences in plasma metabolites across states may play a key role in mediating the enhanced plasma functionalities and inducible immunomodulatory effects observed in the fasted state.

### PF upregulates circulation of multiple immunomodulatory metabolites

**Figure 4:**
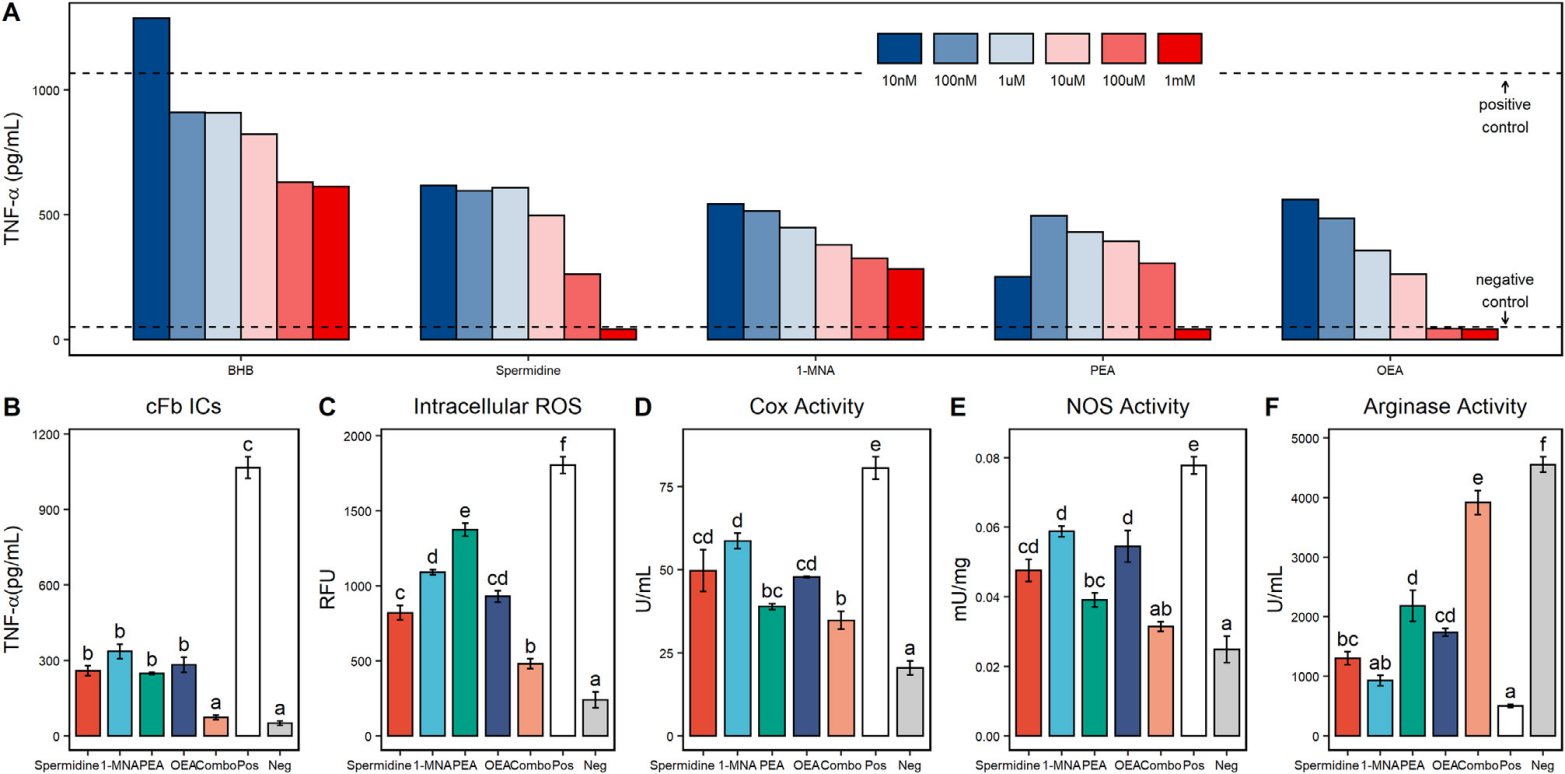
Metabolites upregulated during fasting induce anti-inflammatory effects in macrophage. Tumor necrosis factor alpha (TNF-α) secretion from citrullinated fibrinogen immune complex (cFb IC)-stimulated primary human macrophage treated with beta-hydroxybutyrate (BHB), spermidine, 1-methylnicotinamide (1-MNA), palmitoylethanolamide (PEA), and oleoylethanolamide (OEA) at a final concentration of 1–10 nM (A). The TNF-α levels from unstimulated macrophage are shown as negative control values, and the TNF-α levels from stimulated macrophage without any other treatment are shown as positive control values. TNF-α secretion from cFb IC-stimulated primary human macrophage treated with spermidine (100 μM), 1-MNA (100 μM), PEA (10 μM), OEA (10 μM), and a combination treatment (combo) of all 4 metabolites at their individual concentrations (B). These concentrations were used for all vitro analyses (B–F). The negative and positive control values for all in vitro metabolite analyses (B–F) were generated from unstimulated macrophage or stimulated macrophage with no other treatment, respectively, as described earlier. Significance between treatments and controls for all in vitro analyses (B–F) is denoted by their assigned letter (a–f) where groups that share an assigned letter in common were found to have no significant differences (*P* > 0.05) and groups that do not share an assigned letter in common were found to be significantly different (*P* < 0.05) from each other. Significance was determined using Tukey’s test and is provided for Figure B–F for each treatment group versus the positive control. Positive control, 1065 ± 42.61 pg/mL; spermidine, 259 ± 19 pg/mL, *P* < 0.0001; 1-MNA, 336 ± 29 pg/mL, *P* < 0.0001; PEA, 248 ± 5 pg/mL, *P* < 0.0001; OEA, 283 ± 29 pg/mL, *P* < 0.0001; combo, 74 ± 9 pg/mL, *P* < 0.0001; and negative control, 52 ± 8 pg/mL. Intracellular ROS production from hydrogen peroxide-stimulated primary human macrophage treated with spermidine, 1-MNA, PEA, OEA, and combo (C). Positive control, 1804 ± 55 RFU; spermidine, 820 ± 47 RFU, *P* < 0.0001; 1-MNA, 1091 ± 18 RFU, *P* < 0.0001; PEA, 1375 ± 42 RFU, *P* = 0.0002; OEA, 930 ± 36 RFU, *P* < 0.0001; combo, 481 ± 32 RFU, *P* < 0.0001; and negative control, 241 ± 53 RFU. Total cyclooxygenase (COX) activity from lipopolysaccharide (LPS)-stimulated primary macrophage treated with spermidine, 1-MNA, PEA, OEA, and combo (D). Positive control, 80.6 ± 3.4 U/mL; spermidine, 49.7 ± 6.2 U/mL, *P* = 0.001; 1-MNA, 58.6 ± 2.3 U/mL, *P* = 0.0012; PEA, 38.9 ± 0.9 U/mL, *P* < 0.0001; OEA, 47.8 ± 0.2 U/mL, *P* < 0.0001; combo, 34.7 ± 2.7 U/mL, *P* < 0.0001; and negative control, 20.5 ± 2.1 U/mL. Total NOS activity from LPS- and interferon gamma (INF-γ)-stimulated THP-1 macrophage treated with spermidine, 1-MNA, PEA, OEA, and combo (E). Positive control, 0.076 ± 0.002 mU/mg; spermidine, 0.046 ± 0.003 mU/mg, *P* = 0.001; 1-MNA, 0.059 ± 0.002 mU/mg, *P* = 0.0034; PEA, 0.039 ± 0.002 mU/mg, *P* < 0.0001; OEA, 0.054 ± 0.004 mU/mg, *P* = 0.0009; combo, 0.031 ± 0.001 mU/mg, *P* < 0.0001; and negative control, 0.025 ± 0.004 mU/mg. Arginase activity from LPS- and INF-γ-stimulated THP-1 macrophage treated with spermidine, 1-MNA, PEA, OEA, and combo (F). Positive control, 506 ± 26 U/mL; spermidine, 1032 ± 110 U/mL, *P* = 0.01; 1-MNA, 927 ± 87 U/mL, *P* = 0.1836; PEA, 2181 ± 260 U/mL, *P* < 0.0001; OEA, 1736 ± 68 U/mL, *P* = 0.0007; combo, 3914 ± 203 U/mL, *P* < 0.0001; and negative control, 4550 ± 129 U/mL. All analyses described earlier were performed in duplicate using primary human macrophage isolated from a single healthy donor (A–D) or differentiated THP-1 macrophage (E–F).

To determine the potential plasma-borne mediators of the observed anti-inflammatory effects of PF, we analyzed the dataset of significantly upregulated metabolites between the fasted and baseline states to find metabolites that had previously been shown to have effects on immune cell functionality. Although BHB was highly upregulated during PF (*P* < 0.0001) (see Figure 3C) and, due to its known anti-inflammatory effects, is often thought to be the major mediator of the immunomodulatory effects of PF [40, 41], we also identified numerous other metabolites with known immunomodulatory effects that were upregulated in the fasted state, including dihomo-γ-linolenic acid, succinimide, biliverdin, kynurenic acid, spermidine, 1-MNA, PEA, and OEA. Upon screening of these metabolites at various concentrations in an in vitro model of autoimmune disease as described earlier, we found that 4 of our selected compounds—spermidine, 1-MNA, PEA, and OEA (see Figure 3G–J)—were able to ameliorate TNF-α secretion from stimulated macrophage more potently than treatment with 1-mM BHB at between 100 and 10,000 times lower concentrations (see Figure 4A). Based on these results, we decided to further investigate these 4 metabolites both alone and in combination (combo) in the same in vitro functional analyses that we observed to be affected by treatment with fasted plasma. For each metabolite, we chose a single concentration to be tested based on the half maximal inhibitory concentration (IC50) of each metabolite during initial screening. The experimental results for each analysis are given for the following concentrations: *1*) spermidine, 100 μM; *2*) 1-MNA, 100 μM; *3*) PEA , 10 nM; *4*) OEA , 10 μM; and *5*) combo, 100 μM of spermidine, 100 μM of 1-MNA, 10 nM of PEA, and 10 μM of OEA.

### Fasting metabolites replicate the anti-inflammatory effects of fasted plasma in macrophage

As with fasted plasma, treatment of primary human macrophage with individual compounds or their combination during stimulation with cFb ICs significantly reduced the secretion of TNF-α versus the vehicle-treated positive control (positive control, 1065 ± 42.61 pg/mL; spermidine, 259 ± 19 pg/mL, *P* < 0.0001; 1-MNA, 336 ± 29 pg/mL, *P* < 0.0001; PEA, 248 ± 5 pg/mL, *P* < 0.0001; OEA, 283 ± 29 pg/mL, *P* < 0.0001; combo, 74 ± 9 pg/mL, *P* < 0.0001) (see Figure 4B). Furthermore, the combination of metabolites together reduced the TNF-α levels to a significantly greater extent than the most potent individual metabolite PEA (74 ± 19 vs. 248 ± 5 pg/mL, *P* = 0.019) and was not significantly different from the unstimulated negative control (51 ± 8.7 pg/mL), indicating potent additive anti-inflammatory effects of the metabolites in combination. Similarly, all individual metabolites and their combination were capable of significantly reducing cellular ROS production from primary human macrophage (positive control, 1804 ± 55 RFU; spermidine, 820 ± 47 RFU, *P* < 0.0001; 1-MNA, 1091 ± 18 RFU, *P* < 0.0001; PEA, 1375 ± 42 RFU, *P* = 0.0002; OEA, 930 ± 36 RFU, *P* < 0.0001; combo, 481 ± 32 RFU, *P* < 0.0001), and the level of ROS production in combination-treated cells was significantly reduced beyond the most potent individual metabolite spermidine (481 ± 32 vs. 820 ± 47 RFU, *P* = 0.001) (see Figure 4C). This result was also observed for total COX activity in LPS-stimulated primary macrophage where each individual compound and the combination treatment were able to significantly decrease the total COX activity versus the vehicle-treated positive control (positive control, 80.6 ± 3.4 U/mL; spermidine, 49.7 ± 6.2 U/mL, *P* = 0.001; 1-MNA, 58.6 ± 2.3 U/mL, *P* = 0.0012; PEA, 38.9 ± 0.9 U/mL, *P* < 0.0001; OEA, 47.8 ± 0.2 U/mL, *P* < 0.0001; combo, 34.7 ± 2.7 U/mL, *P* < 0.0001) (see Figure 4D). PEA was a potent inhibitor of COX activity, and combination-treated cells were not found to be significantly lower than those treated with PEA alone (see Figure 4D). These data are in line with previous studies showing that COX activities are influenced by all 4 tested metabolites [[42], [43], [44], [45]] and further underscores the potential of these metabolites to be responsible, at least in part, for the observed COX activity reduction in fasted plasma.

Finally, we found that the in vitro treatment of THP-1 macrophage with individual metabolites and their combination during induced M1 polarization showed a significant reduction in NOS activity (positive control, 0.076 ± 0.002 mU/mg; spermidine, 0.046 ± 0.003 mU/mg, *P* = 0.001; 1-MNA, 0.059 ± 0.002 mU/mg, *P* = 0.0034; PEA, 0.039 ± 0.002 mU/mg, *P* < 0.0001; OEA, 0.054 ± 0.004 mU/mg, *P* = 0.0009; combo, 0.031 ± 0.001 mU/mg, *P* < 0.0001) with a corresponding increase in arginase activity versus the vehicle-treated positive controls (positive control, 506 ± 26 U/mL; spermidine, 1032 ± 110 U/mL, *P* = 0.01; 1-MNA, 927 ± 87 U/mL, *P* = 0.1836; PEA, 2181 ± 260 U/mL, *P* < 0.0001; OEA, 1736 ± 68 U/mL, *P* = 0.0007; combo, 3914 ± 203 U/mL, *P* < 0.0001) (see Figure 4E–F). Decreases in NOS activity were observed to be significantly greater in combo-treated cells for all metabolites except PEA-treated cells (see Figure 4E). Concomitant to blunted NOS activity, combo-treated cells also showed significantly higher arginase activity than those treated with the most potent individual metabolite PEA (see Figure 4F). Ultimately, these findings indicate that treatment of human macrophage with spermidine, 1-MNA, PEA, and OEA, all of which are upregulated in the fasted state, is capable of replicating the immunomodulatory effects induced by fasted plasma. Therefore, spermidine, 1-MNA, PEA, and OEA may be important molecular mediators of at least a portion of the beneficial immunomodulatory effects of PF, especially when used in combination.

### Fasting metabolites extend lifespan in *C. elegans*

**Figure 5:**
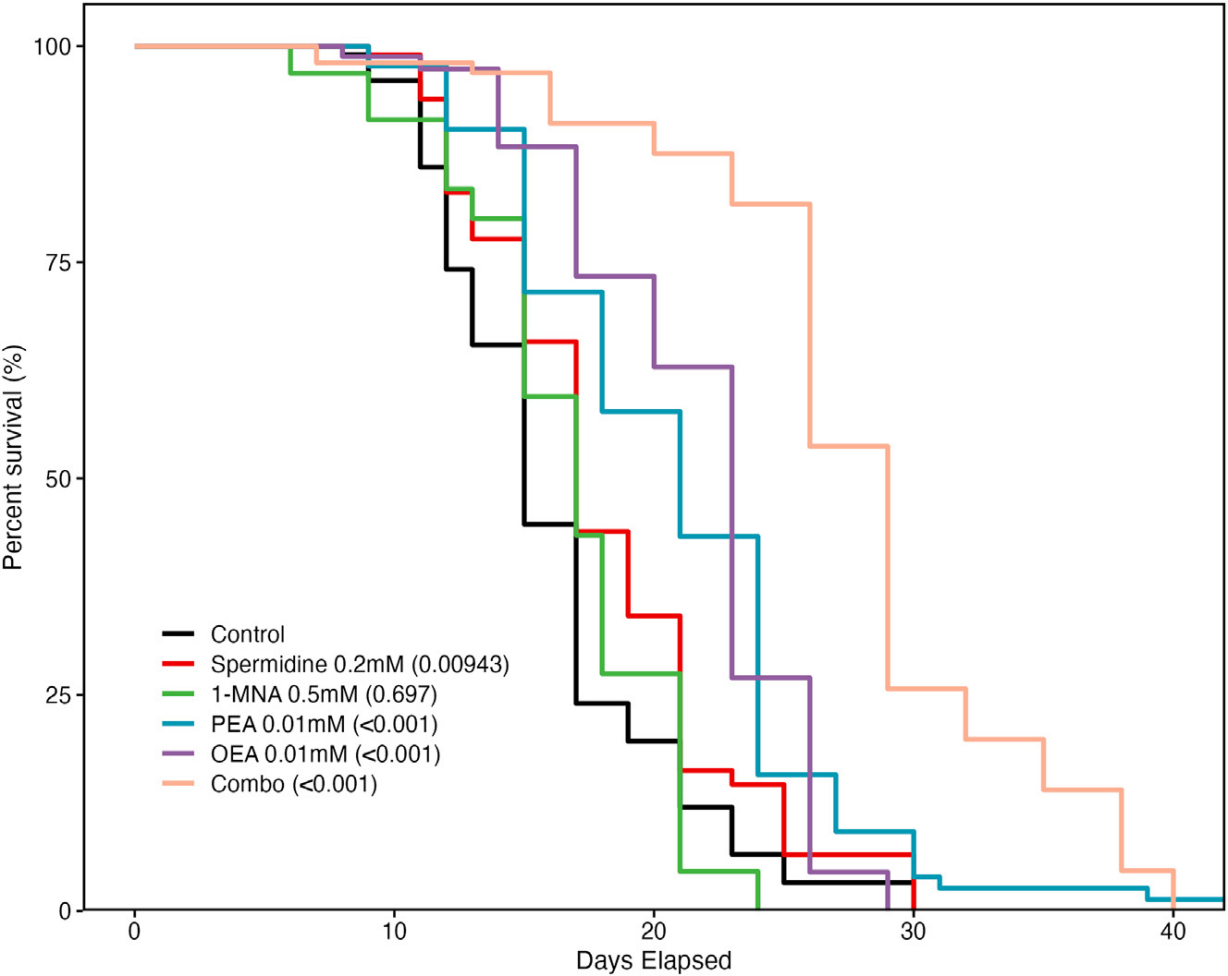
Metabolites upregulated during fasting extend lifespan in *Caenorhabditis elegans*: lifespan analysis of *C. elegans* with either no treatment (control) or lifelong exposure to spermidine (200 μM), 1-MNA (500 μM), PEA (10 μM), OEA (10 μM), or a combination of all 4 metabolites (combo). Significant lifespan extension was observed for the spermidine-, PEA-, OEA-, and combo-treated groups but not for the 1-MNA-treated group—spermidine (*P* = 0.009), 1-MNA (*P* = 0.697), PEA (*P* < 0.001), OEA (*P* < 0.001), combo (*P* < *P* < 0.001). The hazard ratios (HRs) and 95% confidence intervals (CIs) for each treatment group versus the control are as follows: *1*) spermidine, HR, 0.672 (95% CI: 0.498, 0.907); *2*) 1-MNA, HR, 1.061 (95% CI: 0.789, 1.426); *3*) PEA, HR, 0.362 (95% CI: 0.265, 0.493); *4*) OEA, HR, 0.418 (95% CI: 0.304, 0.575); and *5*) combo, HR, 0.147 (95% CI: 0.107, 0.202). The n of each treatment and control group was 120 worms. Significance was determined using a Cox proportional hazards regression model.

Beyond immunomodulation, PF has also been shown to significantly extend lifespan in model organisms [1, 3, 4]. To assess the potential involvement of spermidine, 1-MNA, PEA, and OEA in mediating the lifespan extending effects of PF, we performed lifespan analyses of *C. elegans* whose medium contained individual metabolites or their combination. Strikingly, we found that treatment with spermidine, PEA, OEA, and the combination of spermidine, PEA, OEA, and 1-MNA (combo), but not 1-MNA alone, showed significantly increased lifespan extension versus untreated control worms (see Figure 5). The hazard ratios (HRs) (and 95% confidence intervals [CIs]) for each treatment group versus the control were as follows: *1*) spermidine, HR, 0.672 (95% CI: 0.498, 0.907); *2*) 1-MNA, HR, 1.061 (95% CI: 0.789, 1.426); *3*) PEA, HR, 0.362 (95% CI: 0.265, 0.493); *4*) OEA, HR, 0.418 (95% CI: 0.304, 0.575); and *5*) combo, HR, 0.147 (95% CI: 0.107, 0.202). Of the individual metabolites, PEA and OEA were found to have the greatest effect on lifespan extension at the lowest concentrations and significantly increased lifespan versus spermidine-treated worms (*P* = 0.00943 and *P* < 0.001, respectively) (see Figure 5). Similar to the results seen in vitro, the combo-treated worms showed the highest lifespan extension overall with lifespan being significantly (*P* < 0.001) increased from the control group with a 96% increase to median lifespan and a 51% increase to maximal lifespan. Importantly, lifespan in combo-treated worms was also significantly (*P* < 0.001) increased compared with those in both the PEA and OEA groups, showing a powerful synergistic effect of the combination of these 4 metabolites to extend lifespan. These data suggest that spermidine, 1-MNA, PEA, and OEA are all potential mediators of the lifespan extending effects of PF and treatment with these metabolites even under normal feeding conditions can induce fasting-like benefits to lifespan extension.

## Discussion

In this pilot clinical study, we showed that PF is capable of dramatically altering human plasma functionalities and the plasma metabolome; treatment of nonfasted macrophage with fasted plasma is capable of producing significant anti-inflammatory effects; these effects are mediated, at least in part, by specific bioactive metabolites that are upregulated after 36 h of fasting; and these metabolites are capable of extending lifespan in *C. elegans*.

A key advantage of the current study was the rigorous and controlled design of the human trial. Unlike other studies of fasting, we controlled for dietary intake within individuals using a controlled habitual diet, assessed compliance to fasting through personal glucose monitoring, and collected 4 distinct nutritional states from each individual across the study timecourse. These measures allowed us the ability to sensitively assess differences not only in a prolonged fasted state versus a postprandial state, as most other fasting trials have performed, but also between a 36-h fasted state and an overnight fasted state and postprandial states both before and after 36 h of fasting. This was critical in determining the distinct effects and mediators of PF beyond what is achieved during a typical overnight fast and the carryover effects of PF into the re-fed state without confounding dietary influences.

In this study, PF was capable of significantly increasing plasma antioxidant capacity as well as the cholesterol efflux ability of participant plasma over both the baseline and fed states, and these increases carried over to the re-fed state. Treatment of nonfasted macrophage with fasted plasma also induced powerful anti-inflammatory effects significantly reducing TNF-α secretion, intracellular ROS accumulation, M1 polarization responses, and total COX activity of stimulated human macrophage versus the baseline and fed states. To our knowledge, these are the first studies to show that PF is capable of beneficially modifying human plasma functionalities and treatment of human macrophage with fasted plasma can induce significant anti-inflammatory effects beyond what is achieved by an overnight fast. We are similarly the first to report that PF is capable of ameliorating Fc-γ receptor-induced macrophage activation and reducing total COX activity in macrophage, further elucidating the molecular pathways involved in the immunomodulatory effects of PF. Additionally, although other studies have shown that PF is capable of modulating macrophage polarization in mice [18, 39], we are the first to report that PF is also capable of acutely decreasing M1 polarization responses in human macrophage. These analyses help to underscore that treatment of primary cells with participant plasma during in vitro analyses can induce cellular responses akin to what would be expected in vivo. Thus, such ex vivo modeling can provide sensitive and real-time readouts of systemic functionality throughout the course of a clinical study. Taken together, these findings help to create a clearer picture of the systemic and cellular effects of PF in humans and elucidate new molecular mechanisms underpinning these effects. Significantly, these results also show that not only does PF create anti-inflammatory effects in human macrophage but that these effects are inducible in nonfasted cells via treatment with fasted plasma indicating that the beneficial effects of PF may not be entirely dependent upon cellular energy restriction. Instead, there appear to be multiple bioactive plasma-borne factors that may be responsible for mediating certain cellular responses to PF independent of energy balance.

The importance of investigating these potential plasma-borne mediators cannot be understated as they represent a direct pathway by which the beneficial effects of PF may be replicated without the need to fast. The discovery of such mediators and their development into fasting mimetics represents great potential as therapeutic or preventative interventions for health and disease. In our investigations of these mediators, we showed that the human plasma metabolome in the fasted state versus the baseline state was remarkably altered with 389 metabolites being significantly different between the 2 states. Further analysis and screening of significantly upregulated metabolites in the fasted state revealed spermidine, 1-MNA, PEA, and OEA to have significant anti-inflammatory effects on stimulated macrophage even beyond the effects of BHB, which is often considered to be the major immunomodulatory metabolite during PF [40, 41]. Assessment of these metabolites and their combination showed that all were able to replicate the anti-inflammatory effects observed in fasting plasma including reductions in TNF-α secretion, intracellular ROS accumulation, total COX activity, and M1 polarization responses in stimulated human macrophage. Furthermore, the combination of these metabolites induced the greatest reductions in all these measures compared with any individual metabolite. Thus, the upregulation of these metabolites appears to be responsible, at least in part, for the anti-inflammatory effects observed in fasted plasma. Furthermore, a combination of these metabolites can be used to achieve similar functional effects as PF even in nonfasted cells. To the best of our knowledge, this is the first trial to show that spermidine, 1-MNA, PEA, and OEA are distinctly upregulated in humans during PF. Moreover, although previous studies have shown anti-inflammatory effects for each of these metabolites [[42], [43], [44], [45]], this is the first trial to show their involvement in the immunomodulatory effects of PF on human immunity and their synergistic effect that exceeds the immunomodulatory ability of each individual metabolite.

Finally, we also showed that lifelong treatment of *C. elegans* with spermidine, PEA, OEA, and, most potently, a combination of spermidine, 1-MNA, PEA, and OEA all significantly enhanced lifespan versus control worms even under normal feeding conditions. Previous studies have shown both spermidine and 1-MNA to be able to increase lifespan in *C. elegans* via the induction of autophagy [34, 46] and changes in ROS production, respectively [35]. The mechanisms underlying PEA and OEA lifespan extension are unknown but may be due to effects on the innate immune system, as shown here for humans and which has been implicated in lifespan extension in worms. Thus, it is likely that these compounds affect different and complementary cellular processes to mediate longevity, leading to the potent 96% median lifespan extension observed in combination. These results implicate the importance of these metabolites, and particularly their combination, in mediating the well-known lifespan extending effects of PF and further exemplify the potential for these compounds to be used as fasting mimetics. Significantly, this is also the first study to show that PEA and OEA have lifespan extending effects and, thus, represents the discovery of novel molecules for investigation in longevity research.

In conclusion, the results of this trial identified metabolites and mechanisms of action needed to develop future interventions designed to mimic the effects of PF. This study revealed new functionalities and pathways affected by PF in humans, identified 4 candidates for use as fasting mimetics, showed their effects to be most potent when used in combination, and discovered a previously unknown role of PEA and OEA in lifespan extension. Moreover, this study serves as a strong proof of concept for a human-based approach for the discovery of clinically relevant longevity and health molecules. As with PEA and OEA, by first identifying bioactive molecules in humans and then assessing their roles in longevity extension in model organisms, it may be possible to greatly increase the pace of discovery of longevity targets that could then be immediately translatable to a human health context. Although spermidine has already been investigated for its potential as a caloric restriction mimetic [47, 48], 1-MNA, PEA, and OEA have not, and the effects of combination treatments with different caloric restriction or fasting mimetics are still poorly characterized [48]. It will be a goal for future studies to further clinically investigate the effects of human supplementation with spermidine, 1-MNA, PEA, and OEA both alone and in combination to assess the feasibility and effectiveness of their use in mimicking the effects of fasting.

The authors’ responsibilities were as follows—CR and AZ: conceived of and planned the clinical study design and all experimental analyses performed; CR: performed the clinical study, blood processing, cell isolation and culturing, and in vitro functional assays with assistance from JA; CZ and XT: performed the statistical analysis of the in vitro functional data and metabolomic data; QL and JE: designed and executed the *Caenorhabditis elegans* experiments and performed statistical analysis of the *C. elegans* lifespan data; CR: was the primary author of the manuscript with direction and contributions from AZ and JE: and all authors: read and approved the final manuscript. A patent (PCT/US2021/051104; “Immunological Effects of Metabolites”) related to the work described here has been filed and is currently licensed by UC Davis to Mimio Health, Inc. As part of this license agreement, the University has the potential to receive royalty payments from Mimio Health. CR and AZ have equity interest in Mimio Health, Inc. All other authors report no conflicts of interest.

## Data Availability

Further information and requests for resources and reagents should be directed to and will be fulfilled by the lead contact, Dr. Angela Zivkovic (amzivkovic@ucdavis.edu). This study did not generate new unique reagents. Deidentified clinical data, in vitro experiment data, metabolomic data, and *C. elegans* lifespan data reported in this manuscript will be shared by the corresponding author upon request. This manuscript does not report original code or new standardized datasets. Any additional information required to reanalyze the data reported in this manuscript is available from the corresponding author upon request. All data are available in the main text or the supplementary materials.

## Funding

The clinical study, in vitro functional analyses, and metabolomics analysis were supported in part by a gift grant from the University of California Davis Nutrition Department awarded to CR and by AZ via contributions from National Institutes of Health (NIH; R01AG062240). The *Caenorhabditis elegans* experiments and analyses were made possible by JE via contributions from NIH (GM103860), UC Davis Nutrition Department (CR), National Institute on Aging of the National Institute of Health (R01AG062240) (AZ), and NIH (GM103860) (JE).
