# Supplementary material for: Human fasting modulates macrophage function and upregulates multiple bioactive metabolites that extend lifespan in Caenorhabditis elegans: a pilot clinical study
Source: Am J Clin Nutr. 2022 Dec 20;117(2):286–97. doi: 10.1016/j.ajcnut.2022.10.015 (PMC10196604; doi:10.1016/j.ajcnut.2022.10.015)
Supplement: Multimedia component 1 [file mmc1.docx]

**Title: Human Fasting Modulates Macrophage Function and Upregulates Multiple Bioactive Metabolites that Extend Lifespan in C. elegans, a Pilot Clinical Study**

**Authors:** Christopher H. Rhodes^a^, Chenghao Zhu^a^, Joanne Agus^a^, Xinyu Tang^a^, Qianyan Li^b^, JoAnne Engebrecht^b^, Angela M. Zivkovic^a*^

**Supplemental Figures**


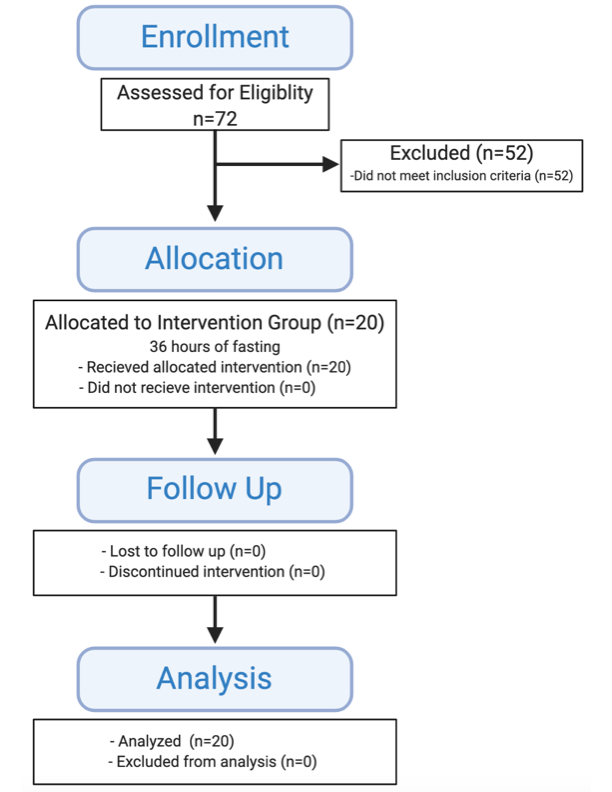


**Supplemental Figure 1.** **CONSORT Diagram Indicating the Structure of the 36hr Fasting Trial:** 72 interested individuals were screened for eligibility and 52 were excluded based on inclusion and exclusion criteria (Supplemental Information). A final 20 participants (10 men and 10 women) were enrolled in the study and underwent the 3-day trial consisting of the intervention of 36hrs of fasting. There were no adverse events, dropouts, or participants lost to follow-up. All 20 participants completed the trial successfully with no protocol violations and were all included in experimental analysis.


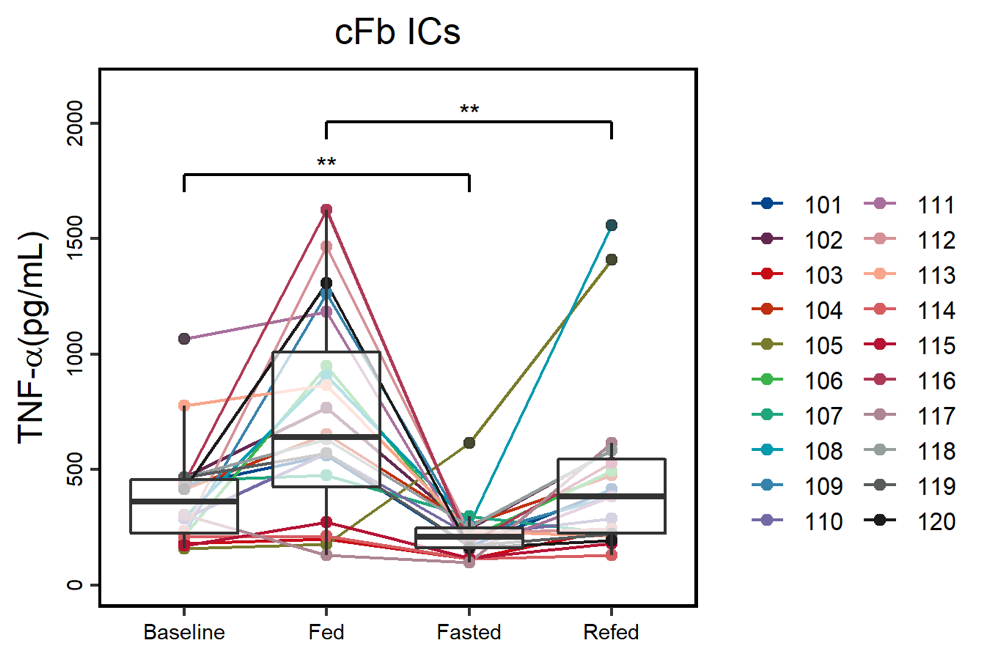


**Supplemental Figure 2. Assessment of Participant Plasma on Macrophage TNF-α Secretion in Alternate Primary Cell Donor:** TNF-α secretion levels from primary macrophage isolated from a secondary healthy male donor and exposed to participant plasma and pro-inflammatory citrullinated fibrinogen immune complexes (cFb ICs). As with the donor cells used for the primary figures there were significant differences between the TNF-α levels in the Baseline vs Fasted states and the Fed vs Refed states.


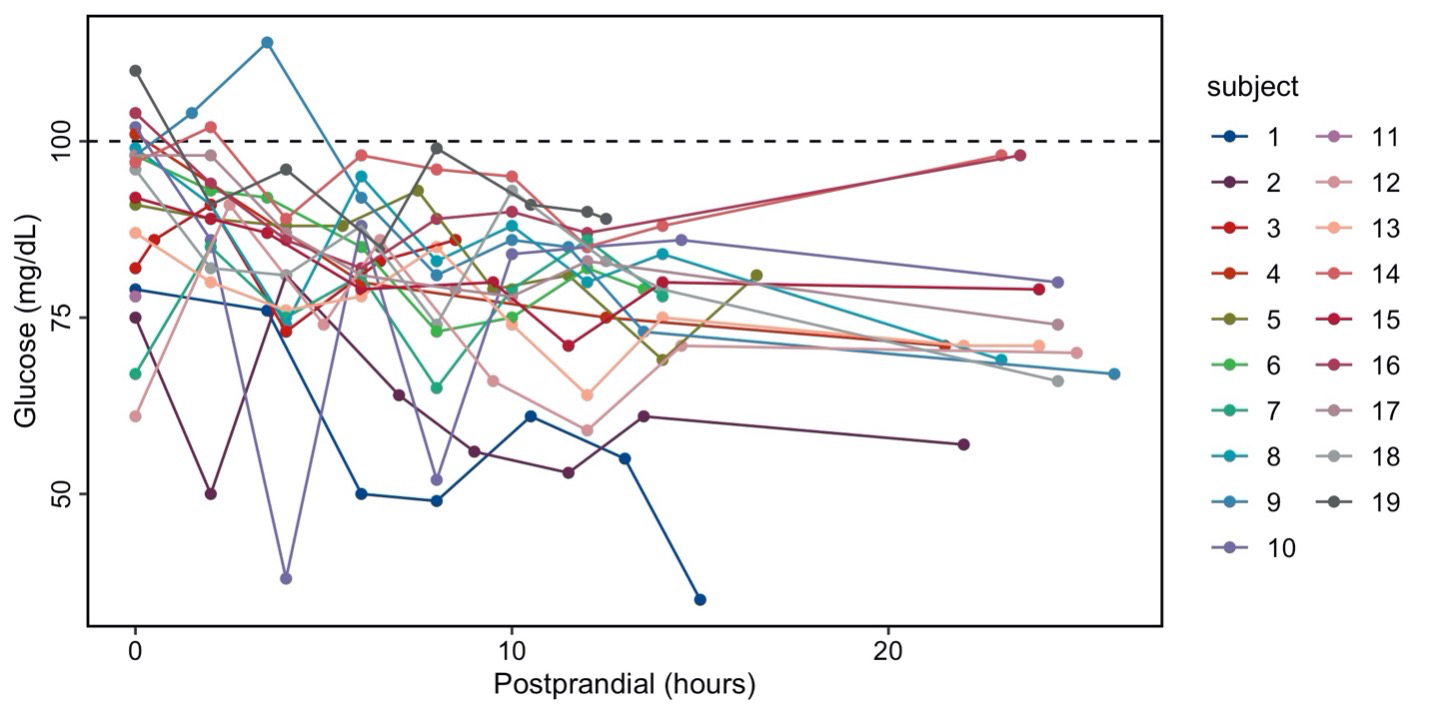


**Supplemental Figure 3.** **Glucose Monitoring Data Indicating Compliance to Fasting:**  Personal glucose monitor values of 20 study participants throughout the fasting timecourse. Glucose readings were taken every 2 conscious hours after the Fed state on Day 1 until the Fasted state blood draw on Day 3. Outside of typical postprandial responses within the first several hours after the Fed state, there were no glucose values above 100mg/dL indicating compliance to fasting.

**Supplemental Table. 1** **Nutrient Intake of Study Participants:**

|  | Baseline (a) | Fed (b) | Refed (d) | pval_ba | pval_da | pval_db |
| --- | --- | --- | --- | --- | --- | --- |
| Cals (kcal) | 2020 ± 673.8 | 1902 ± 564.2 | 1964 ± 553.2 | 0.648 | 0.648 | 0.648 |
| FatCals (kcal) | 741 ± 403 | 683.2 ± 307.9 | 710.7 ± 331.3 | 0.66 | 0.66 | 0.66 |
| SatCals (kcal) | 234.4 ± 178.4 | 182.1 ± 104.9 | 184.4 ± 102.1 | 0.111 | 0.111 | 0.933 |
| Prot (g) | 88.6 ± 43.1 | 79.1 ± 34.3 | 80.5 ± 33.9 | 0.344 | 0.344 | 0.833 |
| Carb (g) | 233 ± 83.6 | 231.5 ± 70.2 | 239.8 ± 68.7 | 0.921 | 0.921 | 0.921 |
| Fib (g) | 27.6 ± 11.7 | 32.3 ± 13.9 | 32.6 ± 13.9 | 0.149 | 0.149 | 0.924 |
| SolFib (g) | 2.3 ± 3.7 | 2.6 ± 2.7 | 2.5 ± 2.8 | 0.837 | 0.837 | 0.837 |
| Sugar (g) | 73.7 ± 40.7 | 70.4 ± 25.7 | 76.3 ± 27.8 | 0.746 | 0.746 | 0.746 |
| MonSac (g) | 13.6 ± 12.5 | 12.5 ± 13.7 | 13.1 ± 14.6 | 0.841 | 0.841 | 0.841 |
| Disacc (g) | 10.3 ± 16.3 | 5.6 ± 5.2 | 5.8 ± 5.6 | 0.215 | 0.215 | 0.955 |
| OCarb (g) | 112.8 ± 63.6 | 110.5 ± 39.8 | 112.7 ± 41.6 | 0.997 | 0.997 | 0.997 |
| Fat (g) | 82.5 ± 45 | 76.1 ± 34.4 | 79.2 ± 37 | 0.66 | 0.66 | 0.66 |
| SatFat (g) | 26 ± 19.8 | 20.2 ± 11.7 | 20.5 ± 11.3 | 0.111 | 0.111 | 0.932 |
| MonoFat (g) | 19.2 ± 17.9 | 18.7 ± 16.2 | 19.7 ± 17.5 | 0.88 | 0.88 | 0.88 |
| PolyFat (g) | 8.2 ± 4.5 | 9 ± 9.1 | 9.5 ± 9.1 | 0.718 | 0.718 | 0.718 |
| TransFat (g) | 0.6 ± 0.8 | 0.2 ± 0.3 | 0.2 ± 0.4 | 0.053 | 0.053 | 0.862 |
| Chol (mg) | 289 ± 247.2 | 235.3 ± 158.2 | 229.7 ± 172.1 | 0.244 | 0.244 | 0.883 |
| Water (g) | 2528 ± 1169 | 2491 ± 1674 | 2595 ± 1667 | 0.906 | 0.906 | 0.906 |
|  |  |  |  |  |  |  |

Average nutrient intake of 20 study participants. Baseline values were determined from 24hr food recalls of the previous day during the Baseline visit. Fed values were determined from food intake recorded throughout Day 1 of the study. Refed values were determined from food intake recorded throughout Day 3 of the study. There were no significant differences observed between any state throughout the course of the study.
